# Effect of a Synthetic Nano-CaO-Al_2_O_3_-SiO_2_-H_2_O Gel on the Early-Stage Shrinkage Performance of Alkali-Activated Slag Mortars

**DOI:** 10.3390/ma11071128

**Published:** 2018-07-03

**Authors:** Bao Liu, Jingbin Yang, Dongxu Li, Feng Xing, Yuan Fang

**Affiliations:** 1 Jiangsu National Synergetic Innovation Center for Advanced Materials (SICAM), Nanjing Tech University, Nanjing 210009, China; baoliu@njtech.edu.cn (B.L.); yangjingbin@njtech.edu.cn (J.Y.); dongxuli@njtech.edu.cn (D.L.); 2 Guangdong Provincial Key Laboratory of Durability for Marine Civil Engineering, College of Civil Engineering, Shenzhen University, Shenzhen 518060, China; xingf@szu.edu.cn

**Keywords:** alkali-activated slag, C-A-S-H gel, drying and autogenous shrinkage, mesopore, microstructure

## Abstract

The relatively high shrinkage of the alkali-activated slag (AAS) has restricted its application as a widely-used building material. This research attempts to study the effect of a hydrothermally synthesized C-A-S-H gel, which has a similar composition to that of the main AAS product, on the shrinkage performance of the AAS. The C-A-S-H nano-particles were mixed into AAS mortars in a proportion ranging from 1 wt % to 5 wt % using two different methods, and the drying and autogenous shrinkage ratio of modified AAS mortars were measured at various ages. The effect of C-A-S-H on their microstructures was also characterized. Results obtained indicated that the addition of C-A-S-H gel to AAS mortars had reduced their drying and autogenous shrinkage, with the addition of 3 wt % reaching the maximum reduction. However, the added amount was not directly proportional to the decrease of shrinkage; the proportion of early-stage drying shrinkage of AAS mortars was greater than that of autogenous shrinkage; the dispersion method was slightly better than the dry mixing method in both shrinkage reduction. MIP results suggested that the addition of C-A-S-H gel had reduced the total porosity and the average pore size of AAS mortars, optimized their pore structure distribution, and significantly reduced the volume of mesopores (<0.05 µm) which resulted in high shrinkage, while the adding method had no significant effect on the pore size distribution of AAS mortars. SEM results showed that the addition of C-A-S-H gel can reduce the crack width of mortars, obtain a dense and uniform matrix structure, increase the density, and effectively suppress both shrinkage deformation of the system, whereas the adding method has no obvious effect on the crack width of the mortar. This research provides a novel approach of the AAS shrinkage reduction and structure refinement, shedding lights on nano-material modification of the AAS.

## 1. Introduction

Alkali-activated material is a new eco-friendly green building material. Using industrial solid wastes (fly ash, slag, steel slag, etc.) as raw materials to react with alkaline activator solution, the preparation process of alkali-activated cementitious material is simple, featured by no firing requirement, low cost, low energy consumption, and small CO_2_ emissions [1,2]. At the same time, the alkali-activated material also has many excellent properties, such as high strength and good durability, thus it has received more and more attention and research in the world. Alkali-activated slag material is a type of cementitious materials that uses slag-type high-calcium-silicon-aluminum as raw material to produce cementitious performance through the alkali-activated process, however, difference in composition and structure of slag may result in great difference on the composition and structure of the hydration products. Among them, one final product is an inorganic cementitious material with a chemical composition similar to the hydration products of Portland cement, and this final product is mainly amorphous calcium silicate hydrate (C-S-H gel); another final product is amorphous calcium aluminate silicate hydrate (C-A-S-H gel). Compared to ordinary Portland cement (OPC), Alkali-activated slag (AAS) has high early and final strength, good frost resistance and high temperature resistance, as well as good corrosion resistance, and reduced risk of alkali-aggregate reaction destruction (compared to the risk of Portland cement), however, the shrinkage of AAS is relatively large [3], and the shrinkage value of AAS is related to the amount of moisture loss. In general, environmental drying and slag hydration are the two main processes leading the internal moisture loss of AAS. As environmental humidity is lower than the humidity inside of AAS, water inside AAS will evaporate, leading to and shrinkage of AAS, a phenomenon often referred to as drying shrinkage [4]. Another process leading moisture loss is slag hydration, which is referred to as self-desiccation, and the corresponding shrinkage is called autogenous shrinkage [5]. In AAS mortar structure, slag hydration and surface drying will occur at the same time. The shrinkage of AAS is the sum of drying shrinkage and autogenous shrinkage. Therefore, the comprehensive studies on drying and autogenous shrinkage of AAS mortar are critical in predicting stresses induced by shrinkage and evaluating risk of cracking.

So far, many researchers have demonstrated that the shrinkage of AAS is considerably higher than that of OPC hardened paste [3,6,7]. For example, Collins et al. [8] found that the high shrinkage of AAS is attributed to the higher capillary stress resulting from the finer pore size distribution (compared to OPC). The research results of Melo et al. [9] revealed that the drying shrinkage of AAS occurs mainly in the early stage of hydration, and increases with the increase of activator; the autogenous shrinkage occurs mainly in the early stage of hardening, and the autogenous shrinkage value is much greater than that of high early-strength OPC. The inherently large shrinkage of AAS leads to its easy cracking characteristic, which is a scientific difficulty in the research of new green cementitious materials. Compared to ordinary Portland cement-based materials, researchers have developed appropriate shrinkage-reducing agents to compensate or reduce the volume shrinkage caused by the hydration process and environmental drying of cement-based materials. Such agents have been proved as effective in practical engineering applications, without causing any side effect of mechanical properties reduction [10,11]. However, there is no appropriately effective method for the shrinkage reduction of AAS cementitious materials (an increasingly popular low-carbon green building material in recent years). The study results of Sakulich et al. [12] have shown that due to its internal curing effect, the use of pre-wet light weight aggregates can reduce the autogenous shrinkage of AAS mortar; Yuan et al. [13] found that the expanding agent is effective for compensating the shrinkage of AAS concrete. Increasing the amount of expanding admixture (EA) results in lowered drying shrinkage of the concrete, Fang et al. [14] showed that MgO can significantly reduce the drying shrinkage of AAS concrete when the content of MgO does not exceed 8 wt %. Currently, there are few reports on the use of shrinkage-reducing agents for OPC in alkali-activated slag cementitious materials, and the shrinkage reduction effect is not good enough. Kalina et al. [15] discovered that polypropylene glycols reduce surface tension and seem to be suitable for shrinkage-reducing admixture (SRA), and the significant reduction of surface tension leads to effective reduction of shrinkage. Bílek et al. [16] found that shrinkage-reducing admixtures (SRAs) based on 2-methyl-2,4-pentanediol exhibits good ability to reduce shrinkage, when 1.0 wt % of this SRA reduced the drying shrinkage of waterglass-activated slag mortar by more than 80%, but at the same time greatly reduced early strengths.

As mentioned above, the main problems impeding the current application of alkali-activated slag cementitious material is that its shrinkage is too large, and the shrinkage-reducing agents used for the conventional Portland cement and its concrete has small or even no effect on AAS. As a result, most AAS studies focused on microstructure and mechanical properties [17,18,19,20,21,22], while the shrinkage characteristics of AAS were barely studied. According to data [23,24], the addition of synthetic C-S-H seeds into OPC can accelerate hydration and reduce porosity, and impose a positive effect on durability. Similar to the role of C-S-H in OPC, C-A-S-H gel acts as the main hydration product of AAS system. By applying the synthesized C-A-S-H gel to AAS, we can investigate the effect of synthetic C-A-S-H gel on the early-stage shrinkage of AAS mortar. In this research, amorphous C-A-S-H gel powder was prepared by hydrothermal method using nano-SiO_2_ and nano-Al_2_O_3_ as raw materials [25]; The powders were then added to the AAS mortar via two different methods. The effect of synthesized C-A-S-H gels on the shrinkage of AAS mortar was studied and a series of tests were performed to determine the autogenous shrinkage and drying shrinkage. The microstructure and reaction products were characterized by SEM-EDS, etc., and MIP tests were conducted to investigate the relationship between mesoporal volume and shrinkage. The conclusions of the research can provide a theoretical basis for the reduction of AAS and promote the study of C-A-S-H gels, and the conclusions can provide a new research approach for further in-depth study on the reduction of alkali-activated cementitious materials.

## 2. Experimental Procedures

### 2.1. Synthesis of C-A-S-H gel and Its Characteristics

**Figure 1:**
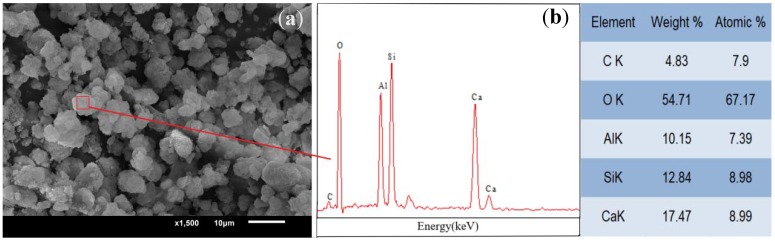
(**a**) SEM image of the C-A-S-H gel; (**b**) Energy-Dispersive Spectroscopy (EDS) results of the C-A-S-H gel.

**Figure 2:**
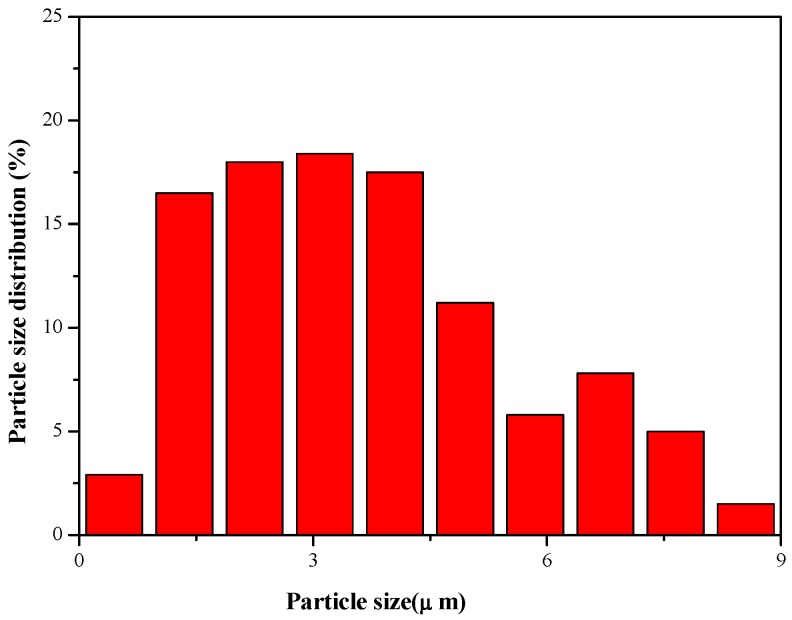
Particle size distribution diagram of the C-A-S-H gel.

This study used nanoscale C-A-S-H gel synthesized according to the hydrothermal method listed in literature [25], with a CaO/SiO_2_ ratio of 1.0 a SiO_2_/Al_2_O_3_ ratio of 2.0, and a water/solid ratio of 8.0. Figure 1 and Figure 2 show the micromorphology and particle size distribution of the synthetic C-A-S-H gel under scanning electron microscope. The synthetic C-A-S-H gel has a flocculent structure, with loose and porous particles as shown in the SEM images; the non-dispersed powder samples are mostly in the range of 2–8 µm in diameter, as shown in Figure 2. The elemental composition of the C-A-S-H gel was analyzed by using EDS, and the average Ca/Si ratio of the gel is close to 1.0. Chemical element compositions of a typical spot were tested by energy dispersive spectrometer, as shown in Figure 1. According to Yang et al. [26], while using hydrothermal method to synthesize low Ca/Si ratio amorphous C-A-S-H gel, the reaction of the raw material Ca(OH)_2_ is complete, and it can improve the early performance of AAS. In addition, the hydration products of AAS are usually C(-A)-S-H gels with a low Ca/Si ratio [27]. Therefore, the synthetic C-A-S-H gel with a Ca/Si ratio of 1.0 was chosen for this research.

### 2.2. Materials, Mixing Proportion and Preparation of Alkali-Activated Slag Mortars

The granulated blast-furnace slag was supplied by Nangang Jiahua New Building Materials Co. Ltd. (Nanjing, China), with a specific surface area of 0.438 m^2^/g and a density of 2.86 g/cm^3^. The chemical composition of slag is summarized in Table 1. The siliceous sand used in this study was obtained from China ISO Standard Sand Co., Ltd. (Xiamen, China). Sodium water glass was used as the alkali-activator. The chemical composition of sodium water glass (produced in Wuxi, Jiangsu Province, China) is SiO_2_ (25.8 wt %), Na_2_O (8.21 wt %) and H_2_O (50.135 wt %), and the molar ratio for SiO_2_/Na_2_O is 1.80. The mix proportions of the alkali-activated slag mortar are listed in Table 2. To determine the water-binder ratio, 15 pre-test teams were selected, with the water-binder ratio ranging from 0.40 to 0.55 at a gradient of 0.1. Considering the flow and reliability, a water-binder ratio of 5.0 was selected in the final study. The specific mixing process used the following two adding methods: ① Dry-mix C-A-S-H gel with the slag (hereinafter referred to as DMS). The C-A-S-H gel powders were evenly divided into three portions, and the first portion was dry- mixed with 30 wt % of the total slag for 3 min, the second portion was dry-mixed with the 30 wt % of the total slag, and the third portion was dry-mixed with the remaining 40 wt % of the slag, with each dry-mixing process lasted for 3 min; ② The C-A-S-H gel was dispersed in water before the mixing by ultrasonic treatment (hereinafter referred to as DW). The C-A-S-H gel powders were evenly divided into three portions, each of which was sequentially dispersed in water and stirred, then each portion went through an ultrasonic treatment for three minutes. Finally, the Sodium water glass solution was added, and the weighed standard sand was poured into the agitator’s sand feeder and stirred in accordance with the procedure of Chinese Standard GB/T 17671-1999. The prepared mortar was cast immediately in a 25 mm × 25 mm × 280 mm triple mold equipped with a copper probe at its end.

### 2.3. Measurements of Length Changes

The formed test piece was cured at standard curing conditions ((20 ± 1 °C), relative humidity greater than 90%) for one day, then the mold was removed, and the initial length L_0_ was immediately measured with a length meter. Four test blocks were measured to obtain the average value, and the measurement accuracy was 0.001 mm. In this research, dry shrinkage refers to the change in length caused by shrinkage due to autogenous drying of mortar and environmental humidity that is lower than the internal humidity, Therefore, the dry shrinkage specimens were placed in a control box with constant temperature (20 ± 1 °C) and constant humidity (relative humidity maintains at 50 ± 3%). In addition, literature [4,9,12,28] showed that autogenous shrinkage is an early part of volume change, which is related to the degree of early hydration. The selection of initial measurement time is an important factor in the evaluation of shrinkage. In this research, autogenous shrinkage refers to the change in length caused by the volume difference between the hydration product and the reactant was measured based on the initial length after 1 day age of curing, that is, the internal self-desiccation shrinkage, under the condition that the mortar sample has no humidity exchange with the outside environment. The autogenous shrinkage is caused by the hydration of mineral powders, and the initial length after 1 day of curing was used as the benchmark. In the test, the parts were cured for 1 day, then removed from the mold and sealed with tin foil to ensure that there was no humidity exchange between the sample and the outside environment. Only the copper head was left exposed, and the change in length at both ends of the copper head was measured and recorded. Place the sample at a temperature of (20 ± 1 °C) for curing, and the relative lengths were measured after different curing time (3 days, 7 days, 14 days, 28 days, 60 days). Shrinkage test was conducted according to Chinese standard JCT 603-2004.

### 2.4. Miscrostructure

The samples for microstructure analysis were taken from the specimens cured in a curing box at different ages. These samples were then immersed immediately in ethanol for 5 days and then dried for 48 h at 60 °C to stop the hydration of AAS mortars.

#### 2.4.1. Mercury Intrusion Porosimetry (MIP)

The total porosimetry and pore size distribution of alkali-activated slag binder at 3, 7 and 28 days of curing were tested by mercury intrusion porosimetry (MIP) (poremaster-60, Quantachrome, Houston, TX, USA). A few samples were crushed into 2–5 mm small pieces and dried at 50 °C for 24 h in a vacuum environment. The pressure of mercury was fixed at 30,000 psi.

#### 2.4.2. Scanning Electron Microscopy (SEM) and Energy-Dispersive Spectroscopy (EDS)

Scanning electron microscopy (SEM) with secondary electron (SE) images was utilized to image the hardened AAS mortars, which can investigate the influence of C-A-S-H on the microstructure characteristics of AAS mortars. Small fractured samples at different hydration ages were respectively dried at 50 °C for 24 h in a vacuum environment. Then the samples were coated with 20 nm of gold to make them conductive. SEM analysis was conducted using a Quanta TM 250FEGinstrument (FEI Company, Hillsboro, OR, USA) with a 20 kV accelerating voltage and a working distance of 10 mm. In addition, scanning electron microscopy (SEM) with backscattered electron (BSE) images and energy dispersive spectroscopy (EDS) analyses were performed to analyze the polished surfaces of the specimens on a NOVA 450 device (FEI Company, Hillsboro, OR, USA). Prior to imaging, specimens were impregnated using a low-viscosity epoxy resin, surface polishing, and carbon-coated.

## 3. Results and Discussion

### 3.1. Effects of Synthetic C-A-S-H on the Shrinkage of AAS Mortars

#### 3.1.1. Drying Shrinkage and Autogenous Shrinkage of AAS Mortars

**Figure 3:**
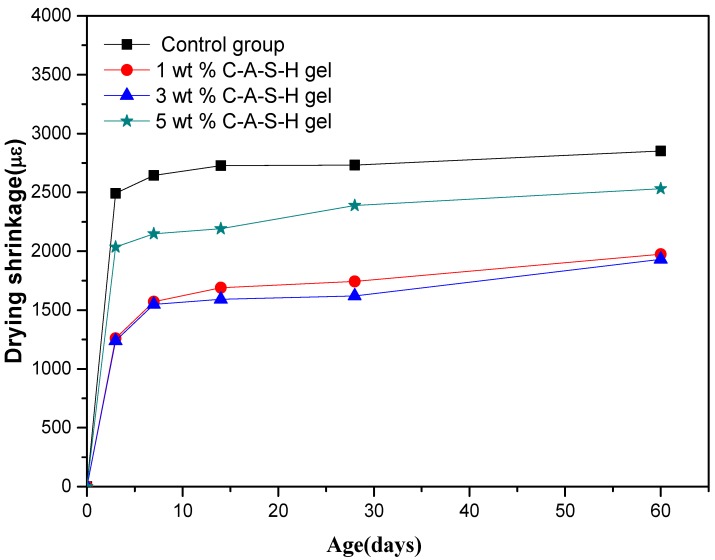
Drying shrinkage of AAS mortars with different amounts of C-A-S-H (dry mix with slag).

**Figure 4:**
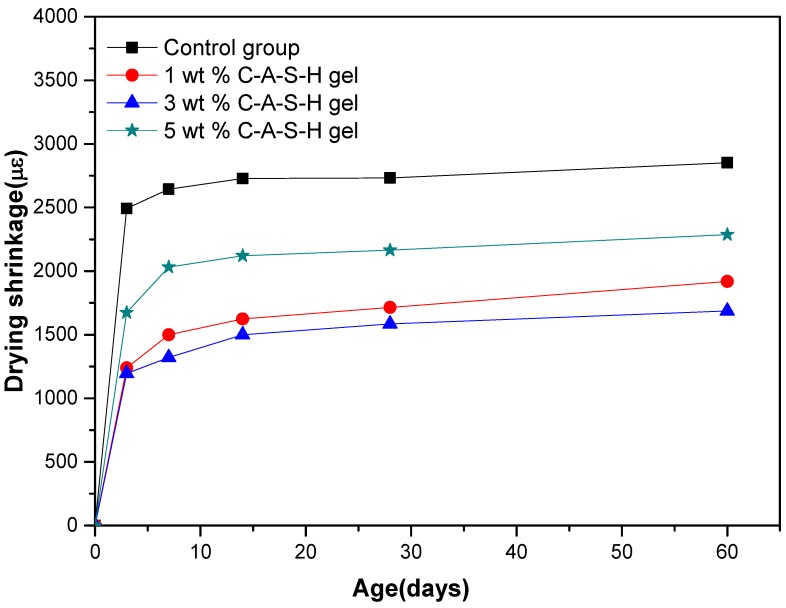
Drying shrinkage of alkali-activated slag (AAS) mortars with different amounts of C-A-S-H (dispersed in water).

Figure 3 shows the effect of different amounts of C-A-S-H gels dry-mixed with the slag on the drying shrinkage of alkali-activated slag mortars. Figure 4 shows the effect of different amounts of C-A-S-H gels dispersed in water on the drying shrinkage of alkali-activated slag mortars. After the addition of the synthetic C-A-S-H gels, the drying shrinkage of AAS mortar was obviously reduced, and the reduction ratio was calculated and summarized in Table 3. 3 wt % C-A-S-H gel brought about the maximum shrinkage reduction (52%), and 7 days were the age period that brought the most significant drying shrinkage of samples.

**Figure 5:**
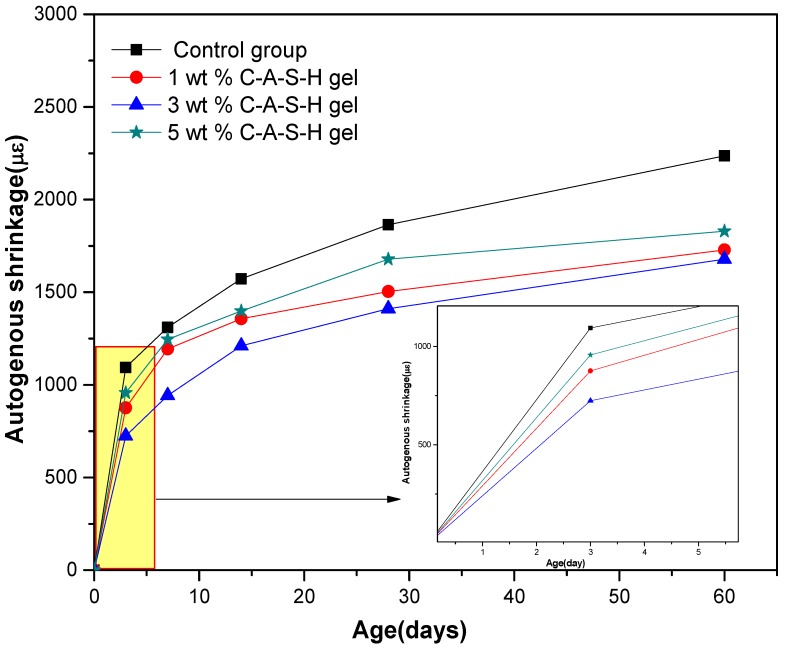
Autogenous shrinkage of AAS mortars with different amounts of C-A-S-H (dry-mixed with slag).

**Figure 6:**
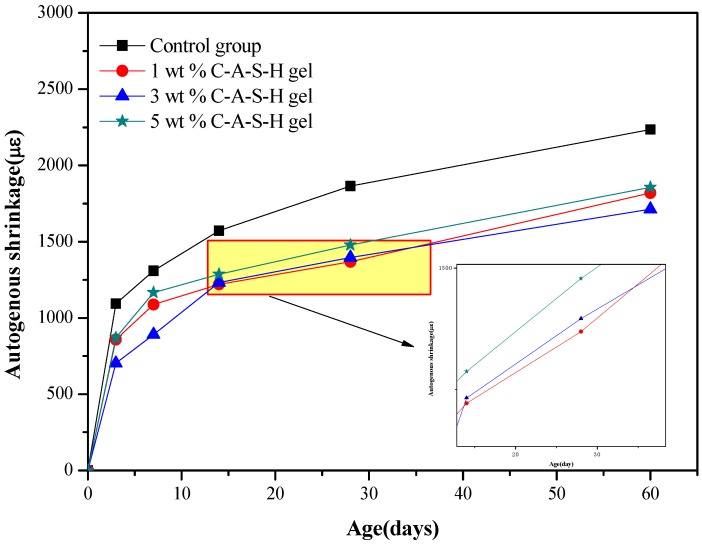
Autogenous shrinkage of AAS mortars with different amounts of C-A-S-H (dispersed in water).

Figure 5 shows the effect of different amounts of C-A-S-H gels dry-mixed with the slag on the autogenous shrinkage of alkali-activated slag mortars. Figure 6 shows the effect of different amounts of C-A-S-H gels dispersed in water on the autogenous shrinkage of alkali-activated slag mortars. After the addition of the synthetic C-A-S-H gel, the autogenous shrinkage of the AAS mortar was apparently reduced, and the reduction ratio was calculated and summarized in Table 4. 3 wt % C-A-S-H gel brought about the maximum autogenous shrinkage reduction (35.74%). In particular, the autogenous shrinkage decreased rapidly after 3 days of age, and such decrease slowed down after 7 days of age.

Combining Figure 3, Figure 4, Figure 5 and Figure 6, it can be seen that the addition of 1 wt %, 3 wt %, and 5 wt % synthetic C-A-S-H gels had a certain shrinkage-reducing effect on AAS mortars, the addition of 3 wt % C-A-S-H gels brought about the most obvious shrinkage-reducing effect, and the shrinkage reduction ratio brought by 5 wt % C-A-S-H gel was smaller than that brought by 3 wt %C-A-S-H gel. According to Yang et al. [26] tests, during the acceleration period, the synthetic C-A-S-H gel was mainly affected by the hydration heat of the alkali-activated slag. The synthetic C-A-S-H accelerates the hydration of the alkali-activated slag and promotes the formation of alkali-activated slag hydration product gels. However, in the synthetic gel test, the required amount of water is pre-added. Larger amount of gel means more water of crystallization, thus the actual of water used in hydration of the slag is reduced, affecting the AAS mortar hydration process. Therefore, there is an upper limit to the content of synthetic C-A-S-H gel, and it is not directly proportional to the reduction of drying shrinkage and autogenous shrinkage.

#### 3.1.2. Relationship between Drying and Autogenous Shrinkage of AAS Mortars

**Figure 7:**
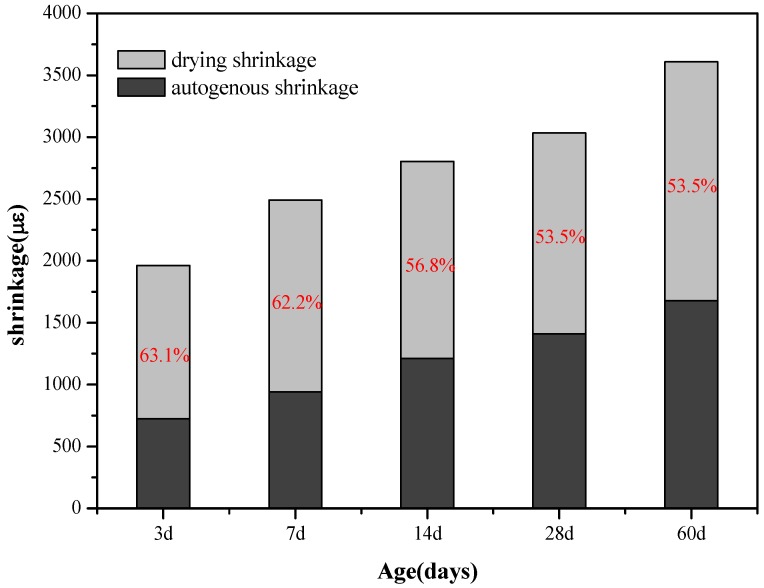
Comparative graph of drying shrinkage and autogenous shrinkage of AAS mortars with the addition of 3 wt % C-A-S-H gels (dry mixed with slag).

**Figure 8:**
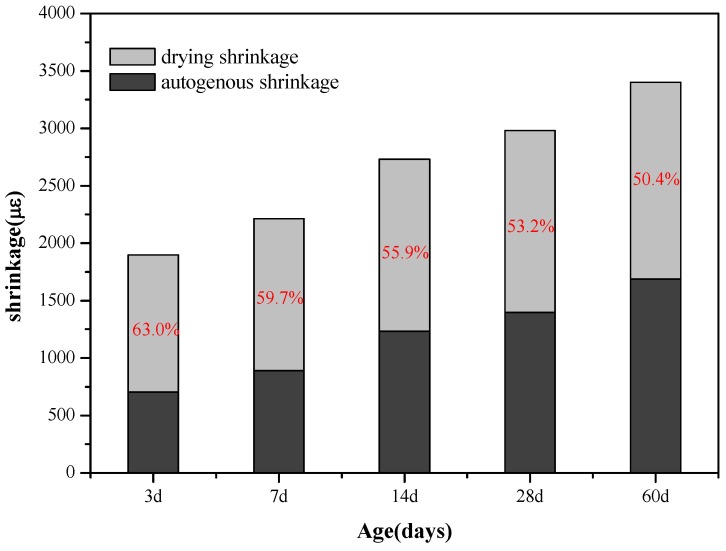
Comparative graph of drying and autogenous shrinkage of AAS mortars with the addition of 3 wt % C-A-S-H gels (dispersed in water).

Figure 7 and Figure 8 show the comparison of the drying shrinkage and autogenous shrinkage of AAS mortars with the addition of 3 wt % C-A-S-H gels through the above-mentioned two mixing methods. The test results showed that the drying shrinkage of all age tests was greater than that of autogenous shrinkage, based on the initial length after 1 day age of curing, and the conclusion is consistent with the research results of Melo et al. [9] and Lee et al. [28]. Specifically, drying shrinkage predominates before 14 days of age, as moisture evaporation in the early stage of the drying process accounts for the most water loss of the mortar, while under sealing condition, moisture loss is mainly due to internal hydration of slag instead of evaporation; however, as the age increases, the proportion of drying shrinkage is significantly reduced, and the drying shrinkage is slightly greater than that of autogenous shrinkage at the age of 60 days. Combined with Figure 3, Figure 4, Figure 5 and Figure 6, after 7 days of age, the drying shrinkage is significantly reduced, because the degree of hydration of the mortar is small at the beginning of drying, and the content of free water is high. All the mortars show large drying shrinkage at the initial stage of drying, however, as the drying time prolongs, the continuous hydration of the mortar will lead to the consumption of free water and the refinement of pore structure, which make it more difficult and slower for the migration and evaporation of water in the pores of the mortar, thus delaying the further development of drying shrinkage. The particle size of the added C-A-S-H gel is between 2 and 8 µm (Figure 2), and a relatively large specific surface area can absorb more free water molecules on its surface, therefore, under sealing condition, the autogenous shrinkage brought by slag hydration gradually slowed down in later stage, and the result is consistent with the research of Cartwright et al. [3].

These results demonstrate that the addition of a proper amount of C-A-S-H gel could reduce both the drying shrinkage and autogenous shrinkage of the AAS mortar under relative humidity. Literature data [8,26,29,30,31] indicate that pore size distribution and characteristics of the hydration products are the key factors affecting the shrinkage of the AAS mortar. In the following text, MIP and SEM-EDS were utilized to investigate the microstructure of hydration products and the structure of AAS mortars, to reveal the influence of C-A-S-H gels from a micro scale. In addition, from Figure 7 and Figure 8 in combination with Table 3 and Table 4, it can be found that the dispersion method is slightly better than the dry-mixing method, and the reason is further explored with the following microscopic characterization.

### 3.2. Effect of Synthetic C-A-S-H on the Pore Structure of AAS Mortars

**Figure 9:**
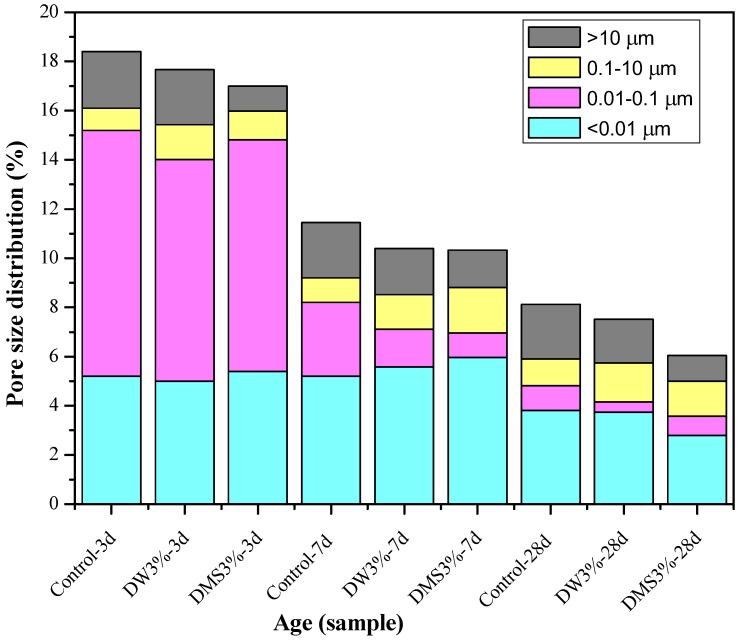
Porosity and pore size distribution of AAS mortars at different curing ages.

Table 5 shows the total porosity, mesopore volume (<0.05 µm)/total pore volume (%), and average pore diameter of the control samples and the samples in different mixing methods with the addition of 3 wt % synthetic C-A-S-H at 3 days, 7 days, and 28 days. Figure 9 shows the total porosity and pore size distribution of AAS mortars. From the data in Table 5, it can be inferred that the addition of an appropriate amount of C-A-S-H gel can reduce the total porosity while reducing the percentage of mesopore volume. The change can also be observed in the average pore size of the slag mortar activated by sodium water glass, as the addition of C-A-S-H gel reduced the average pore size of mortar. From Table 5 and Figure 9, it can be seen that the addition of the synthetic C-A-S-H gel via both mixing methods resulted in different degrees of porosity reduction in the AAS mortar at all three ages. Combining the C-A-S-H gel particle size distribution diagram in Figure 2, it can be seen that because the particle size of the C-A-S-H gel is one order of magnitude smaller than that of the slag particles, one the one hand, it is possible to fill some large pores to increase the internal packing density of the mortar; on the other hand, Zhou et al. [32] believed that the hydration process of AAS is consistent with the OPC system, so the dilution and nucleation effect can be used to explain that the addition of C-A-S-H gel that promotes early hydration of the ore fines, thereby reducing the porosity of the mortar porosity and refining the pore size in the early stage.

As far as the results from MIP and 3.1.1 shrinkage are concerned, from the perspectives of the mortar composition and structure, the shrinkage deformation is mainly caused by the contraction of the porous cemented phase (C-A-S-H gel phase), while the crystalline substances formed by aggregates and hydration generally do not shrink to deform. In all ages, the addition of C-A-S-H gel reduced the percentage of voids of 0.01–0.1 µm and greater than 10 µm and increased the proportion of pores at 0.1–10 µm size range. From Table 5, the percentage of mesopore volume (<0.05 µm) in the total pore volume was reduced. This is probably because the drying shrinkage of AAS mortar is induced by the volume increase of mesopore, and larger mesopore volume can lead to a higher capillary stress in the water meniscus formed in the capillary pores of the mortar, resulting in a higher level of drying shrinkage [3,28,33]. Collins et al. [8] and Tarek Aly et al. [29] also believed that the most important influence on the dehydration and shrinkage deformation in the mortar is the pore structure. The capillary stress caused by the evaporation and migration of water under relative humidity is one of the main causes of the drying shrinkage deformation, therefore, compared to AAS mortars without C-A-S-H gels, AAS mortars added with C-A-S-H gels had a reduced surface tension of pore water, which helps to eliminate pore water [15], reducing the capillary stress that may lead to greater drying shrinkage.

According to research reports [28], the most direct cause of autogenous shrinkage in AAS is the self-desiccation effect. The chemical shrinkage of the AAS after initial curing time leads to the increase of water-free pores in the slag, therefore, self-desiccation will occur in the AAS mortar without external water supply. At the same time, the water in the pores gradually decreases as the hydration reaction continues; the decrease of the relative humidity in the pore system leads to a water-air meniscus, and the resulting capillary stress in the porous structure that exceeds the tension resistance limit of AAS mortar will lead to drying shrinkage [33]. As the mesopore volume (<0.05 µm) of the AAS mortar is associated with the drying shrinkage reduction, and such volume in the mortar added with C-A-S-H gel was much smaller than in the control sample, it can be inferred that the autogenous shrinkage of the AAS mortar is mainly due to the self-desiccation in the hardened state, thus that the extensive drying shrinkage in the sodium water glass-activated slag mortar can be explained by its high proportion of mesopores (<0.05 µm); the water meniscus formed in the capillary pores of the mortar leads to considerable stress on the pore walls, which then turns into significant drying shrinkage, whereas the addition of C-A-S-H gels reduces the proportion of mesopore volume (<0.05 µm), thereby reducing the capillary stress generated in the closed system and accordingly reduces the drying shrinkage.

### 3.3. Effect of Synthetic C-A-S-H on the Microstructure of AAS Mortars

**Figure 10:**
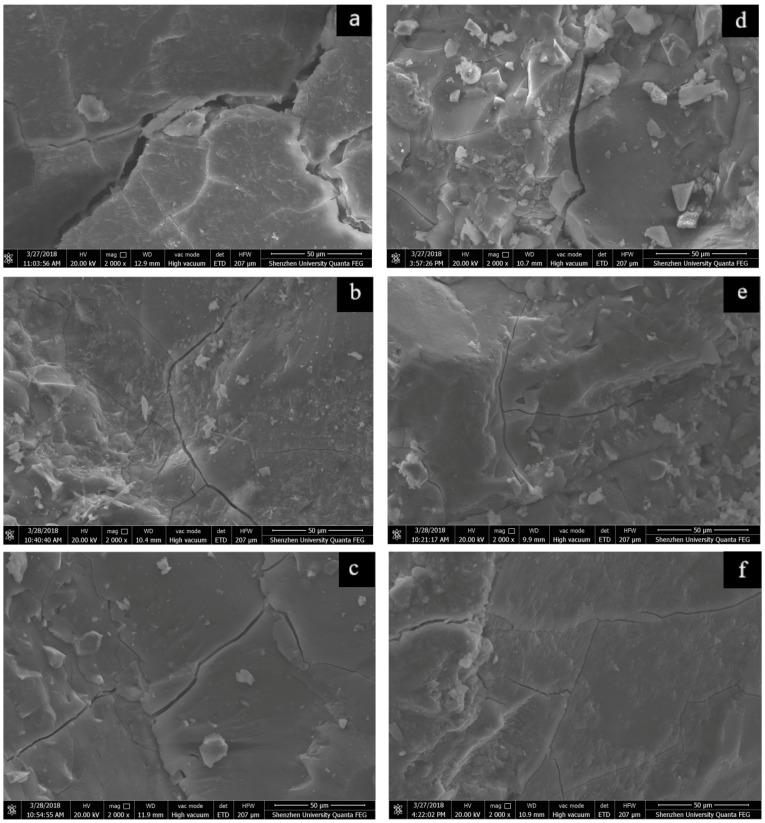
SEM with secondary electron (SE) images of AAS mortars with C-A-S-H gels 3 wt % contents at different ages (**a**) autogenous shrinkage of control sample at 3 days; (**b**) autogenous shrinkage of sample via dispersion method at 3 days; (**c**) autogenous shrinkage of sample via dry-mixed method at 3 days; (**d**) drying shrinkage of control sample at 7 days; (**e**) drying shrinkage of sample via dispersion method at 7 days; (**f**) drying shrinkage of sample via dry-mixed method at 7 days.

SEM analysis and EDS analysis were used to investigate the effect of C-A-S-H gel addition on the microstructure of AAS mortar. Figure 10a–c show the 50 µm microstructure images of the control sample and the AAS mortars added with 3% C-A-S-H gel via two mixing methods after 3 days of autogenous shrinkage. Figure 10d–f show the 50 µm microstructure images of the control sample and AAS mortars added with 3 wt % C-A-S-H gel via two mixing methods after 7 days of autogenous shrinkage. It can be seen that in the absence of C-A-S-H gel, the microstructure of the samples cured at relative humidity shows strong cracks due to significant shrinkage. The structures of the C-A-S-H gel samples are denser than that of the control samples, and the addition of C-A-S-H gel reduces these crack widths. In the electron microscope image analysis software, first draw a line segment of the same length against the 50 µm ruler of the a, b, c, d, e, f pictures, and input the actual length of 50, unit µm, this line segment is the metric value; Then plot a distance on the cracks at different positions in each picture and measure ten different positions to average value, that is measuring the width of the crack. After measurement, the crack width of the control samples (a) at the 3-day age is about 7.41 µm, while the crack width at the 3 days age of the C-A-S-H gel samples (b, c) is about 1.53 µm and 1.58 µm, respectively; The crack width of the control samples (d) at the 7 day age is about 3.11 µm, while the crack widths of samples (e, f) added with C-A-S-H gel at the 7 days age are about 1.16 µm and 1.06 µm, respectively.

**Figure 11:**
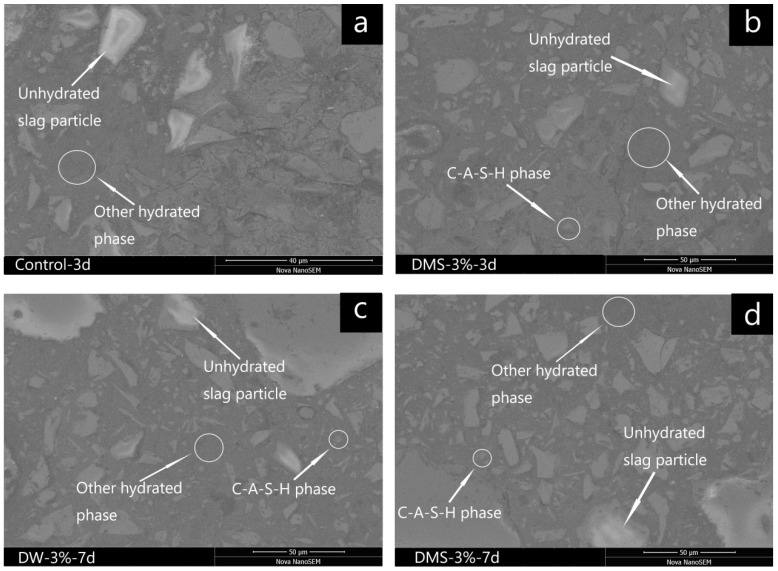
SEM with back-scattered electron (BSE) images of AAS mortars. (**a**) control AAS mortars at 3 days; (**b**) AAS mortars with C-A-S-H gels 3 wt % mixed with slag at 3 days; (**c**) AAS mortars with C-A-S-H gels 3 wt % dispersed in the water at 7 days; (**d**) AAS mortars with C-A-S-H gels 3 wt % mixed with slag at 7 days.

Figure 11 shows the morphology of the control samples and AAS mortars added with 3 wt % synthetic C-A-S-H gel at 3 day and 7 days under a scanning electron microscope with back-scattered electron. Table 6 shows the elemental ratios of these phases as derived from EDS point analyses. Similar to most alkali activated cementitious materials, the hydration products of AAS are mainly gel-like fine particles [34,35], which are microscopic size, disordered structure, variability in composition, and tight binding. From these results in Figure 11 and Table 6, the bright, unhydrated slag particles are all surrounded by grey hydration products, containing calcium-alumina-silicate-hydrate (C-A-S-H) and other hydrated phases. The addition of C-A-S-H gel did not significantly change the phase composition of the hydration products of the AAS mortar, but the difference in microstructure between the control samples and the samples added with C-A-S-H gel can be clearly observed from the graph. As shown in Figure 11a, when the control group curing age was 3 days, many unhydrated slag particles (the gray parts) 5–10 µm in diameter and some pores (the black parts) were observed in the control specimen which did not have a dense matrix structure, and the microscopic appearance demonstrated a looser structure. Compared to the control samples (a), the samples added with C-A-S-H gel (b) had some pores gradually reduce, as indicated in Figure 11b. When the curing period continues to extend to 7 days, the sample added with 3 wt % C-A-S-H gels had fewer pores and a more compact structure, unhydrated slag particles become smaller, as shown in Figure 11c,d. In addition, the morphology at day 7 is clearly smoother than at day 3, because a large number of hydration products form a dense and uniform matrix structure, greatly increasing the density.

To further understand the composition of these products, Table 6 shows the atomic ratios of the reaction products of the specimens by EDS analyses. Previous studies reported that the hydration products of the alkali-activated slag are usually C(-A)-S-H gels with a low Ca/Si ratio [36], the Na/Si ratios of other hydrated phases for the specimens were respectively 0.59, 0.47 and 0.46 on average, as listed in Table 6. This is supported by the presence of Na in other hydrated phases, it can be deduced from these results that a high content of Na was incorporated into the other hydrated phases. Thus, it appears that the Na which dissolved from the sodium water glass activator was more likely to participate in the formation of the C-N-A-S-H gel through the reaction with slag particles, which may be a hybrid type phase of N-A-S-H and C-A-S-H gels [37,38], this is supported by the presence of Na in the other hydrated phase found in the EDS analysis, which corresponds to Lee et al.’s research findings [28]. In addition, as identified from the EDS results, the Ca/Si ratio and Al/Si ratio and chemical composition of the C-A-S-H phase observed by SEM are close to those of the incorporated C-A-S-H gel (Figure 1). It can be seen that a small amount of fine particulate matter is adsorbed on the surface and is bound together with other hydration products nearby, which corresponds to Lloyd et al.’s observation [21].

Another mechanism to explain the modification effect of C-S-H seed crystals on Portland cement is that the added C-S-H gel crystals can greatly reduce the nucleation barrier of the hydration products with its dual roles in adsorption and nucleation [23,24]. Similarly, in the alkali-activated slag system, since the composition and structure of the synthetic C-A-S-H gel and the gel formed by hydration are similar, it can be seen from Figure 10 and Figure 11 that the AAS mortar structure of the control samples is loose and crack width is large, while the AAS mortar structure is gradually compact and the crack width is reduced after the addition of C-A-S-H gel. One possible reason for this is that the surface of the incorporated C-A-S-H gel contains a large number of broken bonds and structural defects, and a higher surface free energy grants C-A-S-H with more capabilities to adsorb ions and molecular. The dual role of adsorption and nucleation changed the hydration process of AAS, so the hydration products obtained a close and uniform structure. Besides, unlike capillary stress, changes in surface free energy can cause overall differential stress in direction, size, and position; capillary stress is more likely to push tighter conical pressures on adjacent solid particles, while surface free energy induces densification of the particles themselves [5]. On the other hand, the body of hardened mortar of AAS material is mainly dense gels [39], and gels are prone to dehydrate, which will cause micro-cracks in the interior of the hardened mortar; the inclusion of gels in the form of particles or strips into the hardened mortar may exert a certain traction control effect on the development of cracks, thereby reducing the crack width.

For the results of MIP and SEM, Figure 9 shows that the mixing method of C-A-S-H gel has no significant effect on the pore size distribution of the mortar; from the crack size comparison in Figure 10b,c,e,f, it can be seen that the mixing method of C-A-S-H gel has no significant effect on the width of cracks. However, as shown in Figure 7 and Figure 8, Table 3 and Table 4, the dispersion method is slightly better than the dry-mixing method. This result can be explained by the report of Land et al. [40], which stated that the dispersion of nanoscale C-A-S-H gels in water using ultrasonic waves can avoid the flocculation of nanoparticles and thus the dispersed particles are more uniform, resulting in a more effective accelerated hydration compared to that of the dry-mixing method. This may be the main reason for the slight difference in shrinkage reduction between the dispersion method and dry-mixing method.

## 4. Conclusions

In this research, a synthetic C-A-S-H with its Ca/Si ratio close to that of AAS hydration products was prepared by hydrothermal method using nanoscale silicon-aluminum as the main raw material. The synthesized C-A-S-H gel was then applied to an AAS mortar to investigate its effect on the early-age drying shrinkage and autogenous shrinkage of AAS mortar. The following conclusions can be drawn from the test results:

(1) The addition of synthetic C-A-S-H gel into the alkali-activated slag mortar can reduce the drying shrinkage and autogenous shrinkage of the AAS mortar. The addition of 3 wt % synthetic C-A-S-H gel reduces the maximum shrinkage, but the increase of C-A-S-H gel content is not directly proportional to the decrease of shrinkage. The proportion of early drying shrinkage of AAS mortar was greater than that of autogenous shrinkage. The dispersion method is slightly better than the dry-mixing method.

(2) MIP results show that the addition of C-A-S-H gel can reduce the percentage of voids between 0.01 and 0.1 µm and greater than 10 µm and increase the proportion of pores between 0.1 and 10 µm at all ages. The addition of C-A-S-H gels can reduce the overall porosity of the AAS mortars, decrease the average pore size, optimize pore structure distribution, and result in a significant reduction in the volume of high shrinkage mesopores (<0.05 µm), which is also the main reason to reduce the drying shrinkage and autogenous shrinkage. The mixing method of C-A-S-H gel has no significant effect on the pore size distribution of the AAS mortar.

(3) SEM results show that the C-A-S-H gel has no significant effect on the phase composition of the AAS product. The main hydration products are amorphous gels. The addition of C-A-S-H gel into the AAS mortar can reduce the crack width of the mortar, obtain compact and uniform matrix structure, increase density, and effectively inhibit the system’s shrinkage deformation. The mixing method of C-A-S-H gel has no obvious effect on the crack width of the mortar.

Our previous studies [25,26] have suggested the synthetic C-A-S-H gel can be regarded as a chemical intensification of the hardening effect in the AAS system, which regulates the kinetics of structure formation in the early stage. The results found in this research further suggests the synthetic C-A-S-H gel could refine the pore structures of the AAS and provide a novel approach of AAS shrinkage reduction at early stage. This shed lights on our future studies as to understand how this nano-gel further effect the microstructure and mechanical performance of AAS system at late stage. Moreover, this gel could be extended to a variety of systems, such as C-(N)A-S-H (CaO-Na_2_O-Al_2_O_3_-SiO_2_-H_2_O), to be further applied to simulate and intensify the alkaline activated materials, including the AAS.

## Figures and Tables

**Table 1:** Chemical analysis of the slag used in this research using XRF (wt %).

CaO	SiO_2_	Al_2_O_3_	MgO	SO_3_	TiO_2_	K_2_O	Fe_2_O_3_	MnO	Na_2_O
39.83	31.13	17.24	8.47	1.2	0.6	0.364	0.234	0.255	0.364

**Table 2:** Mix proportions of the samples (in mass).

No.	Standard Sand (g)	Slag (g)	C-A-S-H Gel (%) (by Slag Mass)	Alkali Equivalent ^a^ (W_(Na2O)_/W_(slag)_) (%)	Water/Slag Ratio
1	1350	450	0	5	0.5
2	1350	450	1	5	0.5
3	1350	450	3	5	0.5
4	1350	450	5	5	0.5

^a^ Alkali equivalent refers to the mass ratio of sodium oxide to slag (sodium oxide is derived from sodium water glass).

**Table 3:** Drying shrinkage reduction of AAS mortars containing synthetic C-A-S-H gels.

Age	C-A-S-H Gels Dosage (wt %)		
	1	3	5
	Shrinkage Reduction (%) Brought by C-A-S-H Gel Dry-Mixed with Slag		
3 day	49.48	50.32	18.30
7 day	40.47	41.41	18.76
14 day	38.05	41.68	19.65
28 day	36.16	40.67	12.55
60 day	30.79	32.30	11.22
	**Shrinkage Reduction (%) Brought by C-A-S-H gel Dispersed in Water**		
3 day	50.24	52.00	32.83
7 day	43.27	50.04	23.18
14 day	40.47	45.01	22.25
28 day	37.26	41.98	20.75
60 day	32.71	40.85	19.81

**Table 4:** Reduction (%) of autogenous shrinkage of AAS mortars containing synthetic C-A-S-H gels.

Age	C-A-S-H Gels Dosage (wt %)		
	1	3	5
	Shrinkage Reduction (%) Brought by C-A-S-H Gel Dry-Mixed with Slag		
3 day	19.01	33.82	12.52
7 day	8.85	28.02	4.89
14 day	15.84	22.96	11.07
28 day	19.31	24.30	9.98
60 day	22.72	24.96	18.20
	**Shrinkage Reduction (%) Brought by C-A-S-H Gel Dispersed in Water**		
3 day	21.48	35.74	20.48
7 day	16.87	31.91	10.92
14 day	22.33	21.63	18.13
28 day	26.56	25.11	20.65
60 day	18.60	23.35	16.99

**Table 5:** Total porosity and average pore diameter of AAS mortars.

C-A-S-H Mixing Method	Mortar	Curing Age	Total Porosity (%)	Mesopore Volume (<0.05 µm)/Total Pore Volume (%)	Average Pore Diameter (µm)
Control	AAS	3	18.40	80.43	0.0226
		7	11.4	70.62	0.0172
		28	8.03	57.84	0.0165
DW	AAS + 3 wt % C-A-S-H	3	17.67	69.23	0.0180
		7	10.4	58.27	0.0163
		28	7.52	45.19	0.0148
DMS	AAS + 3 wt % C-A-S-H	3	16.99	67.57	0.0178
		7	10.33	57.38	0.0161
		28	6.04	47.95	0.0152

**Table 6:** Atomic ratios of different phases by EDS analysis.

Sample	Phase	Ca/Si	Al/Si	Na/Si
		Atomic	Atomic	Atomic
Control	Other hydrated	0.82	0.35	0.59
	Unhydrated slag	1.27	0.43	0.07
DW-3%-7d	Other hydrated	0.88	0.36	0.47
	C–A–S–H	1.00	0.83	–
	Unhydrated slag	1.23	0.41	0.04
DMS-3%-7d	Other hydrated	0.89	0.37	0.48
	C–A–S–H	1.02	0.87	–
	Unhydrated slag	1.25	0.40	0.05
